# HPV Exposure in the Gynecological Practice: Time to Call It an Occupational Disease? A Systematic Review of the Literature and ESGO Experts’ Opinion

**DOI:** 10.3390/vaccines14020148

**Published:** 2026-01-31

**Authors:** Hasan Volkan Ege, Bilal Esat Temiz, Mihaela Grigore, Laura Burney Ellis, Sarah J. Bowden, Belen Lopez-Cavanillas, Mario Preti, Ignacio Zapardiel, Elmar Joura, Murat Gültekin, Maria Kyrgiou

**Affiliations:** 1 Department of Obstetrics and Gynaecology, Division of Gynaecological Oncology, Hacettepe University Faculty of Medicine, Ankara 06230, Turkey; drvolkanege@gmail.com (H.V.E.); bilalesattemiz@gmail.com (B.E.T.); mrtgultekin@yahoo.com (M.G.); 2 Department of Mother and Child, Grigore T. Popa University of Medicine and Pharmacy, 700115 Iaşi, Romania; mihaela.grigore@umfiasi.ro; 3 Department of Obstetrics and Gynecology, Cuza Vodă Hospital, 700038 Iaşi, Romania; 4 Institute of Reproductive and Developmental Biology, Department of Digestion, Metabolism and Reproduction-Surgery and Cancer, Imperial College Faculty of Medicine, London W12 ONN, UKs.bowden@imperial.ac.uk (S.J.B.); 5 Department of Obstetrics & Gynaecology, Imperial College Healthcare NHS Trust, London W2 1NY, UK; 6 Cervical Pathology and Lower Genital Tract Unit, Gynaecology Service, La Paz University Hospital, 28046 Madrid, Spain; belenlopezcavanillas@gmail.com; 7 Department of Surgical Sciences, University of Torino, 10126 Torino, Italy; mario.preti@unito.it; 8 Gynecologic Oncology Unit, La Paz University Hospital, 28046 Madrid, Spain; ignaciozapardiel@hotmail.com; 9 Department of Gynecology and Gynecologic Oncology, Comprehensive Cancer Center, Medical University of Vienna, 1090 Vienna, Austria; elmar.joura@meduniwien.ac.at

**Keywords:** cancer prevention, healthcare workers, human papillomavirus, occupational disease, vaccination

## Abstract

**Background/Objectives:** Persistent human papillomavirus (HPV) infection can lead to malignancies of the cervix, vulva, vagina, penis, anus, and oropharynx. The increasing incidence of HPV-related head and neck cancers has raised concerns regarding potential occupational exposure and transmission risks among healthcare workers. This study aimed to systematically evaluate the evidence on occupational HPV transmission in healthcare settings. **Methods:** A systematic review of the literature was conducted using three electronic databases (PubMed, Scopus, and Web of Science) from inception to August 2025, following PRISMA 2020 guidelines. A total of 34 studies met the inclusion criteria and were included in the review. Expert opinions and practical recommendations from members of the European Society of Gynaecological Oncology (ESGO) Prevention Committee were included to support interpretation of the results. **Results:** The available literature on occupational HPV transmission was limited, with a paucity of high-quality studies. Nevertheless, existing data suggest a potential occupational risk, particularly during aerosol or smoke-generating procedures performed for cervical intraepithelial neoplasia or cervical cancer. Several studies reported the detection of HPV DNA in surgical smoke or on instruments used during such procedures, indicating possible exposure among healthcare workers. **Conclusions:** Although current evidence is insufficient to definitively classify HPV infection as an occupational disease, available data indicate a potential exposure risk for healthcare workers involved in HPV-related procedures. Preventive measures, like personal protective equipment, should be emphasized. HPV vaccination has been recommended by some professional societies for healthcare workers performing gynecological procedures, though further research is needed to evaluate vaccine efficacy beyond the standard age range and its cost-effectiveness in this context.

## 1. Introduction

Human papillomavirus (HPV) is recognized as the most common sexually transmitted infection worldwide [1]. Following transmission, HPV infection typically clears spontaneously in most individuals, but oncogenic HPV infections can persist and lead to cancer. HPV can primarily cause cancers in the cervix, vulva, vagina, penis, anus, and oropharyngeal regions [2]. Outside the anogenital area, HPV-associated cancers are most detected in the head and neck region. The frequency of HPV-associated head and neck squamous cell cancers is increasing, both in the United States (US) and in Europe [3,4]. HPV now accounts for 71% and 51% of all oropharyngeal squamous cell carcinomas (OPSCCs) in the US and United Kingdom (UK), respectively. HPV Type 16 is responsible for more than 80% of HPV-related cancers [4,5].

Despite the fact that the oncogenesis of HPV-related OPSCC resembles the molecular biology of other HPV-associated malignancies, the difficulty in identifying pre-invasive lesions prevents the implementation of screening programmes [2]. Low-risk HPV infections are also implicated in the etiology of laryngeal papillomatosis in the oropharyngeal and laryngo-bronchial regions, which cause significant morbidity [6].

During the treatment of HPV-associated lesions, surgical smoke is generated. Cases of OPSCCs and laryngeal papillomatosis have been reported among healthcare workers, in which transmission through surgical smoke was suggested as a possible etiologic factor. However, evidence regarding the risk of HPV transmission via surgical smoke remains inconclusive. In addition, the potential for HPV contamination through examination instruments or clinical environments has not been clearly established.

HPV is traditionally associated with mucosal transmission; however, increasing evidence suggests that healthcare workers may also encounter HPV through nonsexual occupational exposure routes, particularly during aerosol or smoke-generating procedures.

In this study, we aimed to summarize the available literature investigating occupational HPV exposure and transmission among healthcare workers.

## 2. Materials and Methods

This study was conducted in accordance with the PRISMA (Preferred Reporting Items for Systematic Reviews and Meta-Analyses) guidelines. A systematic literature search was conducted by three independent researchers.

### Search Strategy

The search was performed in September 2025 (including studies published up to August 2025) from inception to present in three databases (PubMed/MEDLINE, Web of Science, and Scopus). Our research keywords were selected to address the question: “Is there an occupational risk of HPV transmission for healthcare workers?”. The occupational exposure concept was operationalized using terms such as “healthcare workers,” “health workers,” “doctor,” “nurse,” “gynaecologist,” “dermatologist,” and “occupational,” each combined with “human papillomavirus.” The complete and reproducible search strategies for all databases, including exact search terms and Boolean operators, are provided in Supplementary File S1. References of the identified studies were also hand-searched to identify additional relevant articles. Only English-language publications were included.

Studies, case reports, surveys, and cross-sectional studies that provided data on occupational HPV exposure among healthcare workers were eligible for inclusion. Additionally, survey-based studies assessing healthcare workers’ knowledge and awareness of occupational HPV transmission risk were also considered. After removing duplicates, all retrieved records were screened by titles and abstracts for relevance. Full-text versions of potentially eligible studies were then reviewed.

Following completion of the systematic literature review and data synthesis, the findings were shared with selected members of the European Society of Gynaecological Oncology (ESGO) Prevention Committee for expert opinion and practical considerations. Experts were selected based on their clinical and academic expertise in gynecologic oncology and HPV-related disease prevention.

## 3. Results

**Figure 1:**
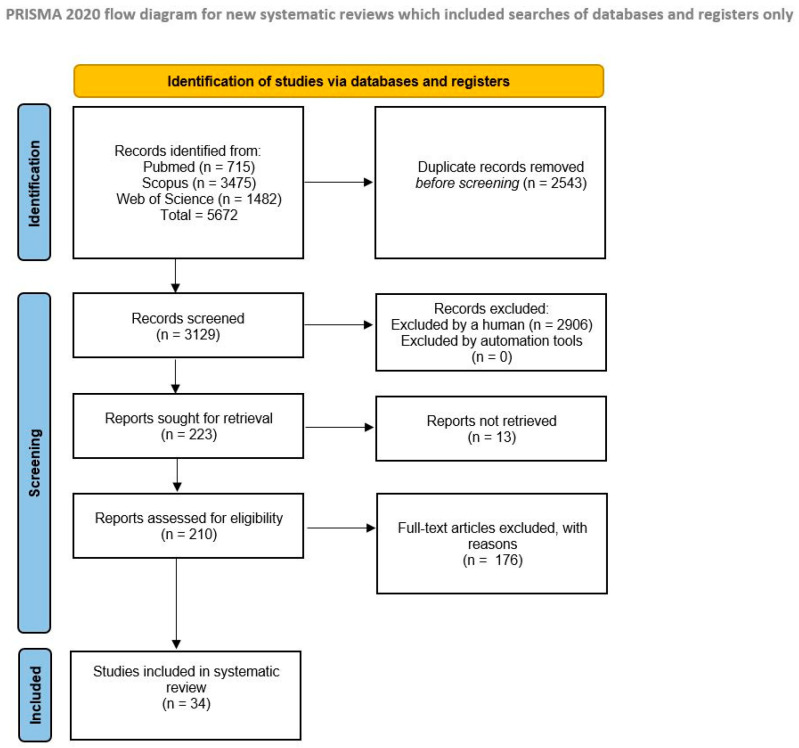
PRISMA flow diagram of the review process.

The search detected 715 articles in PubMed, 3475 in Scopus, and 1482 in Web of Science. One additional relevant case report not indexed in these databases was added manually. After screening and eligibility assessment, a total of 34 articles met the inclusion criteria (Figure 1). Most studies investigated the risk of HPV transmission during surgical procedures involving HPV-related lesions.

The final list of included studies was presented to selected members of the ESGO Prevention Committee for expert discussion. A complete list of included studies is presented in Table 1.

Most studies have investigated the presence of HPV DNA in the surgical smoke generated during the treatment of HPV-associated lesions. In eight studies, HPV DNA was detected either in the surgical smoke collected during procedures or on equipment exposed to the smoke [7,8,9,12,14,20,21,26]. Swabs taken from healthcare workers exposed to smoke during treatment were also analyzed for contamination. In three studies, HPV DNA was found on swabs taken from healthcare workers (including physicians, surgeons, nurses and anaesthesiology staff) from different anatomical sites, such as the oral mucosa, nasolabial fold, nasal cavity and conjunctiva, and on PPE. These findings were suggested as potential evidence of occupational exposure [13,22,25]. Animal studies have also been conducted to investigate the infectious potential of occupational exposure. Garden et al. demonstrated that smoke produced by CO_2_ laser ablation of bovine papillomaviruses (BPV) positive cutaneous fibropapillomas, when applied to the skin of healthy calves, resulted in the development of BPV DNA-positive lesions in all three animals [17]. In another study by Best et al., lesions associated with mouse papillomavirus (MusPV) in mice were treated by sharp excision, KTP laser and coblation. The resulting smoke and sample solutions were applied to mucosally abraded mouse tails, and wart development was observed in all cases [23]. Three studies examined the risk of HPV transmission to healthcare workers through non-aerosol routes. One study investigating HPV DNA contamination on examination equipment (glove box, lamp of a gynaecological chair, gel tubes for ultrasound, colposcope and speculum) found that colposcopes had the highest risk of contamination (OR: 3.02, 95% CI 0.86 to 10.57) [27]. The study demonstrated that HPV contamination could occur despite routine cleaning of gynecological equipment. A prevalence study investigating the incidence of warts among healthcare workers treating HPV-related lesions found that the lesions typically occurred on the hands, highlighting the potential for direct contact transmission [38]. The literature also includes six cases (four case reports) of individuals diagnosed with HPV-related diseases with exposure to surgical smoke during the treatment of HPV-associated lesions [30,31,32,33]. In a case report, laryngeal papillomatosis occurring in healthcare personnel was defined as an occupational disease based on the opinion of a virology institute expert [32]. The risk of aerosol transmission is not limited to procedures that use energy modalities alone. In recent years, studies have suggested that practices such as endotracheal aspiration, intubation–extubation, bronchoscopy, manual ventilation, cardiopulmonary resuscitation, tracheostomy procedures, and high-flow oxygen therapies generate aerosols [41,42].

There are also studies in the literature that do not support the occupational HPV exposure among healthcare workers treating HPV-related diseases. We identified seven studies in which HPV DNA was not detected in the surgical smoke or on equipment used in the treatment of HPV-associated lesions. These studies concluded that the risk of occupational exposure was either absent or minimal [11,15,16,18,19,28,29]. In a 2020 study by Subbarayan et al., no HPV DNA was detected in the smoke produced from the cauterization of mouse tails injected with DNA plasmids. The same study also failed to detect HPV DNA on PPE or robotic arms used during robotic oropharyngeal cancer surgeries [24]. There are also observational studies with control groups addressing this issue. In a survey conducted by Kofoed et al. involving 287 participants, although the swabs from healthcare workers who performed treatments on HPV-related lesions showed higher HPV positivity, the difference was not statistically significant compared to the control group [40]. Similarly, in a study by Gloster et al. involving 570 surgeons, no significant difference was found in the prevalence of warts between those who performed CO_2_ laser treatments and those who did not perform CO_2_ laser treatments (5.4% vs 4.9%; *p*: 0.56) [39]. There was also no significant difference in PPE use (e.g., gloves, masks, smoke extraction) between the warts and non-warts groups.

In four questionnaire-based studies, the perceptions of healthcare workers involved in the treatment of HPV-related lesions regarding the risk of occupational HPV exposure were evaluated. In the study conducted by Leahy et al., which included 75 healthcare professionals treating cutaneous lesions, 36.8% of participants acknowledged the risk of HPV infection through surgical smoke, and 76.8% reported concern about potential occupational exposure [34]. In another study by Al-Dawsari et al., 55.7% of healthcare workers reported developing warts following dermatologic procedures [35]. Afsar et al. assessed both awareness of the risk of occupational exposure and attitudes towards HPV vaccination among healthcare workers [36]. In this study, 84% of participants were aware of the risk of occupational HPV exposure and expressed willingness to receive the HPV vaccine if available. In a survey by Mercier et al., it was found that while many participants expressed concern about occupational exposure to HPV, they had not received sufficient education on the risk [37]. Notably, approximately one-third of adult vaccinations reported in the study were motivated by concerns over occupational HPV exposure. All four surveys demonstrated that health care workers were concerned about potential infection while treating HPV-related lesions.

## 4. Discussion

According to the Protocol of 2002 to the Occupational Safety and Health Convention, 1981, the term “occupational disease” covers any disease contracted as a result of exposure to risk factors arising from work activity. In the identification of an occupational disease, two fundamental elements are discernible: firstly, the causal relationship between exposure in a specific working environment or work activity and a specific disease; and secondly, an incidence rate among exposed workers that is higher than that in the general population [43]. This framework raises the question of whether HPV infection should be considered an occupational disease. Sexual transmission is the most well-known and documented mode of HPV infection. However, fomites, non-sexual contacts such as mouth-to-finger or skin contact, contribute to HPV transmission [44]. It is possible for the patient to infect themselves through fomites or fingers (oral infection and auto-inoculation). Vertical transmission from mother to the baby through different routes has been demonstrated [45]. Studies examining HPV transmission in gynaecological examination areas have documented HPV contamination in instruments used [27,46]. Traditional disinfection methods may not be effective in eliminating HPV transmission in medical equipment and devices [44]. Resistance shown by HPV to commonly used chemicals such as glutaraldehyde or alcohol supports evidence of transmission from fomites and surfaces. All of these findings can support the existence of nosocomial infection risk for HPV, in particular, in healthcare facilities where sterilization with high-temperature or high-pressure autoclaves is not employed [46].

Transmission of HPV infection via aerosol and non-aerosol is a currently debated issue. Certain clinical procedures appear to confer a higher occupational risk of HPV exposure, particularly those that generate surgical smoke or aerosols. Procedures such as electrocautery, laser ablation, LEEP, and other energy-based interventions used in the treatment of HPV-related lesions have been frequently associated with the detection of HPV DNA in surgical plumes. These risks are especially relevant for healthcare professionals in gynecology, otolaryngology, dermatology, and head and neck surgery, where prolonged or repeated exposure to smoke-generating techniques is common. During procedures performed for HPV treatment, surgical smoke contains various toxic materials, both biological and non-biological, and poses a risk of HPV transmission through aerosols [7,12,13,14,17,18,19,20,21,22,23,25,26]. Additionally, the risk of occupational HPV transmission during excisional and ablative procedures was comprehensively evaluated in the systematic review and meta-analysis in 2021, which demonstrated detectable HPV DNA in surgical smoke and documented variable exposure risk for healthcare workers [6]. Measures to be taken to protect against surgical smoke, which is potentially infectious, can reduce HPV exposure (such as proper aspiration during electro-surgical procedures generating smoke, use of N95 masks) [8,9,13,14,17,19,22,23,25]. However, there are studies in the literature that do not support the transmission of HPV through surgical smoke [15,16,24]. Differences in HPV DNA detection techniques, sampling methods, and analytical sensitivity may partly explain the heterogeneity of results across studies.

Case reports have documented instances of laryngeal papillomatosis, tonsillar cancer, oropharyngeal cancer and tongue carcinoma linked to HPV occupational exposure [30,31,32,33]. Although case reports cannot establish a causal relationship, they should be considered as important signals that may indicate potential occupational risk. Several studies in the literature indicate that healthcare workers are concerned about the risk of occupational HPV exposure [34,35,36,37]. However, these studies also demonstrate that healthcare workers generally lack sufficient knowledge regarding preventive strategies for occupational HPV exposure [37]. Despite the presence of many identified and estimated modes of transmission, a professional risk definition for healthcare workers has not been made for HPV infections [47]. Healthcare workers have not yet been included in high-risk groups for HPV infection, even though not only gynaecologists but also many other healthcare groups may be at risk of HPV transmission [48].

Despite existing concerns and reports of HPV-related disease cases, there are still obstacles to defining HPV infection as an occupational disease. Comprehensive studies on the incidence of HPV infection among healthcare workers need to be conducted to investigate whether there is a significant difference compared to the general population. Additionally, determining the actual source and route of transmission in a healthcare worker with HPV infection poses another challenge. The limitations of screening and follow-up programs for HPV infection and related diseases further complicate the issue. Currently, healthcare workers undergo periodic screenings for certain infectious agents depending on the centres in which they work, but there are no applicable periodic screening programs for HPV (except for cervical diseases at present). While more evidence may be needed, based on the available literature, necessary precautions should still be taken regarding the risk of HPV transmission among healthcare workers. Possible measures to reduce occupational HPV exposure among healthcare workers are summarized in Table 2.

### 4.1. Role of HPV Vaccines

The HPV types prevented by the 9-valent vaccination account for approximately 90% of HPV-attributable cancers worldwide [49]. In 2020, the U.S. Food and Drug Administration approved GARDASIL-9 (Merck & Co., Rahway, NJ, USA) for boys and men for the prevention of anal, oropharyngeal, and other head and neck cancers caused by HPV types 16, 18, 31, 33, 45, 52, and 58. International randomized controlled trials conducted with female adolescents and women aged 15 to 26 have demonstrated a vaccine efficacy of at least 96% in preventing cervical precancers (cervical intraepithelial neoplasia grade ≥ 2 or adenocarcinoma in situ) caused by the specific HPV types targeted by the vaccine. [50] Trials of the quadrivalent vaccine demonstrated 100% efficacy in preventing anogenital warts [51,52].

Although cervical cancer screening programmes are widely implemented, vaccination remains the only effective strategy for preventing other HPV-related diseases, such as head and neck cancers, for which routine screening is far less effective and cannot be recommended [53,54,55]. However, there is still a lack of robust, literature-based evidence of a significant reduction in oral HPV 16–18 infections after bivalent vaccination, particularly in males [56].

The American Society for Colposcopy and Cervical Pathology (ASCCP) has recommended that healthcare professionals responsible for gynecological procedures, such as the excision or ablation of lesions associated with HPV, should receive the HPV vaccine. This recommendation extends to healthcare workers engaged in gynecological operations, including nurses, physicians, nurse practitioners, dermatologists, anesthetists, otolaryngologists, family practice, gynecologic oncology, pulmonologists, critical care medicine, and health technicians, who are also at risk and could benefit from expanding vaccination guidelines [57]. The American College of Obstetrics and Gynecologists reports that it concurs with ASCCP’s recommendation, despite the lack of sufficient data supporting the efficacy or cost-effectiveness of the vaccine [47]. The British Association of Dermatologists recommends using HPV smoke aspirators, PPE, further occupational health research, and professional training to prevent disease transmission via smoke plumes, including HPV aerosolization during procedures but did not mention vaccination [58]. In a December 2022 statement, the World Health Organization (WHO) stated that vaccination recommendations for healthcare workers are the same as for the general population [59]. The WHO does not categorise HPV vaccines as recommended for healthcare workers [60]. The National Institute for Occupational Safety and Health has established recommendations for locations where medical procedures generating smoke are performed [61]. In recent years, numerous articles have highlighted the need to evaluate HPV vaccination and the use of PPE for healthcare workers due to potential occupational exposure [47,62,63,64,65,66].

Several studies have investigated HPV vaccination rates among healthcare workers in recent years. The results of these studies showed that vaccination rates varied, ranging from 7% to 41% [36,67,68,69,70,71]. A study evaluating the vaccination habits of medical students found that 32% were fully vaccinated, while 15% were partially vaccinated [72]. Cost or limited availability have been found to be some of the main reasons cited for not getting vaccinated [67,71].

However, population-level vaccine effectiveness appears lower with increasing age [73]. Although the vaccine is approved for individuals up to 45 years of age, it is less effective after the age of 26, which highlights the importance of early vaccination [74]. Therefore, HPV vaccination should ideally be administered as early as possible during a healthcare professional’s career, particularly before the onset of occupational exposure. In fact, vaccinating future healthcare workers who are at risk of exposure to HPV early in their training (e.g., during the first years of medical school or nursing school) may present an important opportunity. Additionally, catch-up vaccination or educational programs within medical schools could help increase HPV vaccine uptake among those who missed it during adolescence.

However, vaccination may still be considered later in life for healthcare workers with ongoing or anticipated occupational risk. In the context of procedures associated with aerosol or surgical smoke generation, HPV vaccination may offer potential occupational benefit and should be discussed irrespective of age, following appropriate individual counseling.

Importantly, the reduced population-level effectiveness of HPV vaccination in older individuals primarily reflects prior HPV exposure rather than diminished vaccine-induced immune responses [75].

Although HPV vaccines have demonstrated an excellent overall safety profile, careful monitoring for adverse reactions following initial doses remains essential [75]. This is particularly important when vaccination is offered outside standard age-based programs.

Healthcare institutions and healthcare leaders may have a unique opportunity to improve HPV vaccine uptake by integrating vaccination into existing occupational vaccination programs, such as seasonal influenza or Hepatitis B vaccination campaigns. Targeting healthcare workers under the age of 46 during routinely scheduled institutional immunization sessions could represent a practical and cost-effective strategy to increase coverage among individuals at potential occupational risk.

We found there was a lack of large-scale observational studies examining the incidence of HPV related diseases in healthcare workers versus the general population. Such studies are of course still subject to bias but would inform both the strength of the association and the types of occupational exposure most likely to result in HPV-related disease.

### 4.2. Strengths and Limitations

To our knowledge, this systematic review provides one of the most comprehensive overviews of HPV-related occupational exposure, incorporating both recent evidence and healthcare professionals’ perspectives on risk perception and preventive practices. We combine this systematic review with expert opinion from across Europe. The review captures both supporting and opposing evidence for HPV occupational transmission, incorporating studies from diverse medical disciplines. Recommendations regarding PPE, smoke evacuation, and vaccination are concrete and actionable.

The article has some limitations. Firstly, most studies supporting the association between HPV transmission to healthcare workers and treatment procedures are either animal studies or involve a small number of participants. The risk of bias in the literature is therefore high. The authors acknowledge the limited evidence base, highlighting methodological constraints and a lack of large prospective studies. While the survey results show awareness and concern among healthcare workers, they cannot establish risk causality as perceived risk (including psychological issues) is different from biological risk. Further limitation is the slow progression of HPV infection, which obscured the identification of the incident HPV infection as an occupational exposure.

Interpretation of occupational HPV exposure is also inherently limited by the high background prevalence of HPV in the general population and by the fact that routine screening is restricted to cervical disease. These factors make it difficult to distinguish occupational acquisition from prior or non-occupational exposure.

## 5. Conclusions

Lesions related to HPV infections are frequently treated, and healthcare workers are at risk of HPV transmission during these procedures. Moreover, the evidence suggests that some interventions and procedures on individuals with HPV virus carriers, even in cases where HPV-related disease treatment is not performed, have the risk of transmission through contact or aerosol. While the evidence base was limited, the likelihood of this risk should prompt the consideration of HPV infection in healthcare workers as an occupational disease, and this designation should be confirmed with high-quality prospective studies. We recommend consideration of implementing the following preventive strategies due to the potential occupational risk of HPV infection: well-equipped intervention areas/operation rooms (containing smoke evacuators or room suction systems), PPE to reduce the probability of disease (surgical mask, gloves, etc.) and administration of HPV vaccines in populations with proven effectiveness.

Research priorities should include not only seroprevalence studies but also prospective cohort studies focusing on HPV-related disease outcomes among occupationally exposed healthcare workers, along with molecular studies assessing HPV DNA in surgical smoke using standardized detection methods and cost-benefit analyses of extending HPV vaccination to healthcare workers.

## Figures and Tables

**Table 1:** Summary of studies.

Author	Year	Participants	Findings
Studies examining the risk of HPV transmission to healthcare workers by aerosol route (n = 20)			
Garden JM [7]	1988	7 patients	HPV DNA was studied in smoke from the treatment of papillomavirus-infected warts, and intact HPV DNA was detected in smoke from the treatment of 2/7 (28.5%) cases.
Sawchuk WS [8]	1989	8 patients	HPV DNA was studied in smoke from plantar wart treatment. Five of eight laser-derived vapors and four of seven electrocoagulation-derived vapors were positive for human papillomavirus DNA. The authors indicated that the use of surgical masks may protect operators from potential inhalation exposure to papillomavirus.
Ferenczy A [9]	1990	110 patients and 1 surgeon	No post-procedure HPV DNA has been detected in samples from surgeons treating HPV-containing genital lesions (nasopharynx, eyelids and ears). HPV DNA was detected in 1/5 (20%) prefilter canister. The authors indicated that the use of appropriate equipment to evacuate HPV DNA-positive smoke would eliminate the risk of contamination.
Wisniewski PM [10]	1990	Not available	Airborne viral transmission by laser debris during treatment of HPV-associated lesions is unlikely. Improvements are needed in surgical smoke evacuation.
Abramson AL [11]	1990	7 patients	HPV DNA was not detected in surgical smoke when the tip of the aspirator was not in contact with infected tissue. HPV DNA was found in some of the aspiration materials collected by contacting the tissue.
Kashima HK [12]	1991	22 patients (19 RRP, 3 non-RRP)	HPV DNA (type 6 or 11) was detected in 17 of 27 smoke samples from 19 patients with recurrent respiratory laryngeal papillomatosis who underwent Carbon dioxide (CO_2_) laser excision. The authors indicated that healthcare workers (particularly endoscopic surgeons and surgical teams) exposed to laser vapor would be at potential risk of HPV transmission.
Bergbrant IM [13]	1994	79 samples taken from surgeons	Samples (from the nostrils, nasolabial folds, and conjunctiva) taken by the surgeon treating HPV-related genital lesions with CO_2_ laser or electrocoagulation were tested. HPV DNA was positive in 7 of the pre-treatment samples and in 15 of the post-treatment samples. The authors mention that CO_2_ laser or electrocoagulation procedures carry a risk of HPV transmission and that air evacuation with the use of masks is required in the treatment area.
Sood AK [14]	1994	49 patients	HPV DNA was detected in 39% (n: 18) of the smoke samples collected from 49 patients who underwent Loop electrosurgical excision procedure (LEEP) with a diagnosis of cervical intraepithelial neoplasia (CIN). The authors mentioned the need to use gloves, masks and effective smoke evacuation methods to reduce the potential risk of infection to patients, surgeons and other operating room personnel.
Kunachak S [15]	1996	10 samples	Smoke collected from CO_2_ laser-irradiated laryngeal papilloma samples was inoculated into cultures. No evidence of viral infection was found in the cultures. The authors concluded that CO_2_ laser treatment of laryngeal papillomavirus-infected cells with a specific power density (equal or more than 1667 W/cm^2^) prevented airborne transmission.
Hughes PSH [16]	1998	5 patients	HPV DNA was not detected in the smoke from warts treated with the Erbium YAG laser (Continuum Biomedical, Dublin, Calif.). All warts were positive for HPV DNA. The authors suggest that erbium: YAG laser treatment of warts is safe in terms of HPV exposure.
Garden JM [17]	2002	Animal model	Smoke (containing papillomavirus DNA) collected by laser exposure of cutaneous fibropapillomas was inoculated into calf skins. Tumor development at the inoculation sites supported that smoke exposure could cause disease. The authors recommend the use of aerosol-producing lasers with the correct indication and the use of smoke extraction systems and personal protective equipment (PPE).
Weyandt GH [18]	2011	20 samples	During treatment of HPV-associated lesions with argon plasma coagulation (APC), HPV DNA was detected in samples taken from the surgeon’s eyeglasses and nasolabial fold swabs in a subgroup that differed from samples taken from patients. The authors argued that APC carries a low risk of HPV transmission to operating room personnel.
Ilmarinen T [19]	2012	18 Healthcare Workers (120 samples)	The transmission of HPV to healthcare workers during the treatment of laryngeal papillomas and urethral warts was investigated. While HPV DNA was detected in gloves (genital wart group 10/10 and RRP group 4/10), HPV DNA was not detected in oral mucosa or surgical mask swabs. The authors concluded that the use of masks, gloves and goggles would help protect healthcare workers from HPV infection during the treatment of laryngeal papilloma and genital warts.
Akbarov I [20]	2013	66 patients	HPV DNA was investigated in the smoke obtained in the treatment of genital warts using YAG-Laser. HPV DNA was detected in all 66 cases.
Neumann K [21]	2018	24 patients	HPV DNA (same subtype as the subtype detected in the excision material) was detected in 4 of the surgical smoke samples collected from 24 patients who underwent LEEP for high-grade squamous intraepithelial lesion (HSIL).
Zhou Q [22]	2019	31 Healthcare Workers, 134 patients	A significant correlation was observed between the presence of HPV DNA in surgical smoke and HPV infection in cervical cells. Analysis of nasal swab samples collected from 31 gynaecologists performing LEEP procedures revealed consistency in the distribution of HPV subtypes in two operators, as detected in corresponding cervical cell and surgical smoke samples. Those operators with positive nasal swabs for HPV 16 or 58 were determined, through regular examinations conducted every 3 months over the subsequent 35 and 43 months, to be free from HPV-related diseases such as warts. Additionally, it is noteworthy that LEEP operators with detected HR-HPV in nasal swabs were found to wear ordinary masks, potentially indicating lower efficacy in preventing viral infections compared to N95 masks.
Best SR [23]	2020	Animal model	Healthy mice were exposed to smoke samples or contaminated instruments used during treatment of mice with papillomavirus-associated lesions. All mice developed warts. The authors recommend that healthcare workers take appropriate preventive measures to reduce the risk of infection during the treatment of papillomavirus-associated diseases.
Subbarayan RS [24]	2020	Animal model and 3 patients	HPV DNA was not detected in the surgical smoke generated by the cauterization of a mouse tail injected with plasmid DNA expressing HPV p16, E6, and E7 genes. Similarly, no HPV DNA was detected in surgical smoke, suction tubing, the surgeon’s mask, or the robotic arm during robotic surgery for oropharyngeal cancer. The authors reported that the risk of occupational exposure to HPV16 from electrocautery smoke is likely to be minimal.
Hu X [25]	2021	700 Healthcare Workers	HPV DNA was analyzed in nasal swab samples from physicians treating HPV-related lesions, with 67% in the electrosurgery group and 33% in the non-electrosurgery group. HPV DNA detection was significantly higher in the electrosurgery group compared to the non-electrosurgery group (8.96% vs. 1.73%; *p* < 0.001). The incidence of HPV was lower among those who wore surgical masks during procedures (7.64% vs. 19.15%). Notably, no HPV DNA was detected in individuals using N95 masks, whereas HPV positivity was significantly higher among those using standard surgical masks (0% vs. 13.98%; *p* < 0.001). The use of smoke-absorbing devices did not significantly reduce HPV incidence. No cases of HPV-related malignancies or diseases were identified in the study cohort.
Yan L [26]	2022	18 patients	HPV DNA was detected in 83% (n = 15) of cases in the surgical smoke generated by ultrasonic scalpel use during cervical cancer surgery. The authors reported that surgical smoke is an occupational risk for healthcare workers.
Studies examining the risk of HPV transmission to healthcare workers via the non-aerosol route (n = 3)			
Gallay C [27]	2016	179 samples from equipment	Samples collected from gynecological examination areas, including glove boxes, gynecological chairs, lamps, ultrasound gel tubes, colposcopes, and speculums, revealed an overall HPV positivity rate of 18%. Colposcopes had the highest contamination rate (OR: 3.02, 95% CI: 0.86–10.57).
Dodhia S [28]	2018	12 patients	HPV DNA was investigated in potassium titanyl phosphate (KTP) laser fibres used in the treatment of respiratory papillomatosis. HPV DNA was not detected in any of the KTP laser fibres.
Lucet JC [29]	2019	676 TVS procedures	Transvaginal ultrasound probe (TVS) and ultrasound keyboard were investigated for the presence of hrHPV. No hrHPV DNA was found in probe swabs, while 0.3% positivity was found in the keyboard. Despite inadequate compliance with hygiene guidelines, no evidence of hrHPV DNA was found in TVS probes.
Case reports with evidence of HPV-associated occupational disease (n: 4)			
Rioux M [30]	2013	2 Healthcare Workers	Patient A, a 53-year-old gynaecologist, developed HPV16-positive tonsillar squamous cell carcinoma after performing more than 3000 laser ablations and LEEPs over a period of more than 20 years, with no other risk factors. Patient B, a 62-year-old gynaecologist with a 30-year history of performing similar procedures, developed HPV16-positive base of tongue cancer with minimal other risk factors. These cases suggest that HPV transmission through laser plume may lead to squamous cell carcinoma.
Hallmo P [31]	1991	1 Healthcare Worker	The surgeon who performed anogenital condyloma treatment was diagnosed with laryngeal papillomatosis. The detection of HPV 6 and 11 in the lesion was interpreted as a result of inhaled virus particles present in the laser plume.
Calero L [32]	2003	1 Healthcare Worker	A 28-year-old gynaecological operating theatre nurse was diagnosed with laryngeal papillomatosis, which had been found several times during the treatment of anogenital condylomas. This clinical condition was associated with occupational exposure.
Parker J [33]	2021	2 Healthcare Workers	Patient 1, a gynecologist with remote minimal smoking history and a 31-year monogamous relationship, developed SCC after 27 years of treating ~250 HPV-related lesions without mask or smoke evacuation. Patient 2, a 66-year-old gynecologist, lifelong nonsmoker in a 40-year monogamous relationship, developed SCC after treating ~500 lesions over 40 years, using only a surgical mask. Although plume exposure from HPV lesion treatment may be a risk factor for OPSCC, causality remains unproven.
Opinions of healthcare workers about occupational exposure in the treatment of HPV-associated lesions (n: 4)			
Leahy M [34]	2023	75 Healthcare Workers	In Ireland, a survey among healthcare professionals, including dermatologists, dermatology trainees, nurses and general practitioners who encounter and treat HPV-related lesions, revealed that only 36.8% of respondents were cognizant of the risk of contracting HPV infection through procedures emitting aerosols/smoke, such as cryotherapy, electrocautery, and CO_2_ lasers. However, 76.8% expressed concerns regarding the occupational transmission of HPV.
Al-Dawsari NA [35]	2021	228 Healthcare Workers	Among the study participants, 34.6% were found to be infected with HPV, and during the dermatological practice, 55.7% of these individuals manifested the development of warts.
Afsar S [36]	2024	349 Healthcare Workers	Among participants (in USA), 84% were aware of occupational HPV exposure and associated risks. Additionally, 90% expressed willingness to receive the HPV vaccine if provided. However, only 30% were aware of the ASCCP recommendation regarding HPV vaccination for healthcare personnel involved in HPV-related treatments.
Mercier AM [37]	2024	37 Healthcare Workers	One-third of HPV vaccinations administered during adulthood have been reported to be motivated by concerns about occupational HPV exposure. The study concluded that healthcare workers lack sufficient knowledge regarding occupational HPV exposure.
Studies comparing health care workers treating HPV-associated lesions with control groups (n: 3)			
Lobraico RV [38]	1988	794 Healthcare Workers	The rate of verrucous lesions was 3.2% (n:26) among healthcare workers who treated verrucous lesions with laser. The highest rate was found in dermatologists of 15.2%. A total of 1.7% of gynaecologists and 1.6% of paediatricians reported having lesions. The fact that most lesions occurred on the hand suggests direct contact transmission. The absence of lesions on the buccal mucosa or larynx suggested that the risk of viral contamination from laser smoke was insignificant.
Gloster HM Jr [39]	1995	570 Healthcare Workers	Surgeons who used CO_2_ laser and surgeons who did not use CO_2_ laser had similar rates of warts (5.4% vs 4.9%; *p*: 0.56). Nasopharyngeal warts were significantly more common in the laser-used group. There was no significant difference in the use of gloves, standard surgical masks, laser masks, smoke evacuators, eye protection, or full surgical gowns between the wart and non-wart groups.
Kofoed K [40]	2015	287 Healthcare Workers	Oral rinses and nasal swabs were collected from dermato-venerology and gynaecology staff. Mucosal HPV positivity was 5.8% in the experienced laser treatment of the genital warts group and 1.7% in the inexperienced group (*p*: 0.12). It was reported that the prevalence of HPV did not increase significantly among healthcare workers who performed treatments for genital warts or cervical dysplasia.

**Table 2:** Occupational preventive measures against HPV exposure in healthcare workers.

Risk Source/Procedure	Recommended Protective Measure
Surgical smoke (electrocautery, laser, LEEP)	Smoke evacuation systems, N95/FFP2 masks
Aerosol-generating procedures	Adequate ventilation, PPE
Contaminated instruments/surfaces	High-level disinfection and sterilization
Prolonged occupational exposure	HPV vaccination
Lack of awareness or knowledge	Education and awareness programs

## Data Availability

The data supporting the findings of this systematic review are included within the article and its Supplementary Materials. All analyzed data were derived from previously published studies cited in the reference list.

## Supplementary Materials

The following supporting information can be downloaded at: https://www.mdpi.com/article/10.3390/vaccines14020148/s1, File S1: The keywords used in the literature search and the search results.

## Abbreviations

The following abbreviations are used in this manuscript:

ESGO	European Society of Gynaecological Oncology
HPV	Human Papillomavirus
US	United States
UK	United Kingdom
OPSCCs	Oropharyngeal squamous cell carcinomas
CO_2_	Carbon dioxide
CIN	Cervical intraepithelial neoplasia
LEEP	Loop electrosurgical excision procedure
PPE	Personal protective equipment
HSIL	High-Grade Squamous Intraepithelial Lesion
SCC	Squamous Cell Carcinoma
ASCCP	American Society for Colposcopy and Cervical Pathology
BPV	Bovine papillomaviruses
WHO	World Health Organization
