# Supplementary material for: HPV Exposure in the Gynecological Practice: Time to Call It an Occupational Disease? A Systematic Review of the Literature and ESGO Experts’ Opinion
Source: Vaccines (Basel). 2026 Jan 31;14(2):148. doi: 10.3390/vaccines14020148 (PMC12945036; doi:10.3390/vaccines14020148)
Supplement: Supplementary file 1 [file vaccines-14-00148-s001.zip › File S1.pdf]

Systematic Search in Databases

No Date Limit	PubMED	WOS	Scopus
healthcare workers [Title/Abstract] AND human papillomavirus [Title/Abstract]	84	99	205
health workers [Title/Abstract] AND human papillomavirus Title/Abstract]	129	240	601
gynaecologist [Title/Abstract] AND human papillomavirus [Title/Abstract]	28	328	848
dermatologists [Title/Abstract] AND human papillomavirus [Title/Abstract]	54	51	211
doctor [Title/Abstract] AND human papillomavirus [Title/Abstract]	172	262	630
nurse [Title/Abstract] AND human papillomavirus [Title/Abstract]	175	442	760
occupational [Title/Abstract] AND human papillomavirus [Title/Abstract]	73	60	220
**Total**	**715**	**1482**	**3475**
